# Skin-Test Screening and Tuberculosis Transmission among the Homeless^1^

**DOI:** 10.3201/eid0811.020306

**Published:** 2002-11

**Authors:** Po-Marn Kong, Jan Tapy, Patricia Calixto, William J. Burman, Randall R. Reves, Zhenhua Yang, M. Donald Cave

**Affiliations:** * Denver Public Health, Denver, Colorado, USA; † University of Colorado Health Sciences Center, Denver, Colorado, USA; ‡ Regional Tuberculosis Genotyping Laboratory, University of Arkansas for Medical Sciences, Little Rock, Arkansas, USA; § Central Arkansas Veterans Healthcare System, Little Rock, Arkansas, USA

**Keywords:** homelessness, tuberculosis, tuberculin skin testing, DNA fingerprinting

## Abstract

We describe the implementation of a mandatory tuberculosis (TB) screening program that uses symptom screening and tuberculin skin testing in homeless shelters. We used the results of DNA fingerprinting of *Mycobacterium tuberculosis* isolates to evaluate the effect of the program on TB incidence and transmission. After the program was implemented, the proportion of cases among homeless persons detected by screening activities increased, and the estimated TB incidence decreased from 510 to 121 cases per 100,000 population per year. Recent transmission, defined by DNA fingerprinting analysis as clustered patterns occurring within 2 years, decreased from 49% to 14% (p=0.03). Our results suggest that the shelter-based screening program decreased the incidence of TB by decreasing its transmission among the homeless.

Homelessness is one of the greatest risk factors for contracting tuberculosis (TB) in the United States (1,2). The incidence in the general population is extremely low; however, the incidence among the homeless, particularly among minority ethnic groups, can be as high as 450 cases per 100,000 population (3). In a previous study, homelessness was the key factor associated with DNA fingerprint clustering, suggesting that ongoing transmission of TB in Denver, Colorado, occurs primarily among the homeless (4).

Several studies suggest that active case finding may decrease transmission and the overall case rate (2,5,6). We describe the implementation of a mandatory screening program consisting of symptom screening and tuberculin skin testing in homeless shelters and evaluate its effect on TB incidence and transmission.

## Methods

### Description of Screening Program

The Denver Metro TB Clinic of Denver Public Health Department provides TB control activities for metropolitan Denver, Colorado, which includes the counties of Adams, Arapahoe, Denver, Douglas, and Jefferson. Active TB is treated by use of directly observed therapy in the clinic or in the community; outside the clinic, outreach workers deliver the therapy using incentives and enablers (i.e., transport, food coupons, temporary housing, and treatment for substance abuse or mental illness). Temporary housing for the homeless in Denver is available in large communal shelters, through residential drug and alcohol treatment programs, and in individual or family transitional housing. Our program focused on the large communal shelters and the drug and alcohol treatment programs because these settings were the likely loci of TB transmission.

The screening program included a simple symptom assessment and a tuberculin skin test for persons who had no history of a positive skin test. The symptom assessment was a brief interview to detect TB symptoms such as chronic cough, weight loss, night sweats, and hemoptysis.

We used the Mantoux method of tuberculin skin testing (6) and defined a positive result as induration of >10 mm. Patients with symptoms that suggested active TB (regardless of tuberculin skin-test results) and those with a previous or current positive skin test were sent to the Denver Metro TB Clinic for further evaluation and chest radiograph. To stay in a homeless shelter or residential substance abuse treatment program for more than 3 to 5 days, patients were required to complete the screening. All persons with active TB were treated with directly observed therapy, and persons with latent TB infection who met criteria for treatment were offered directly observed preventive treatment. After completion of screening, homeless persons were given a card from the clinic that allowed them to remain in one of the shelters for up to 6 months.

The screening program, started on a voluntary basis in 1993 at a day shelter and a clinic for the homeless, was expanded to four large shelters and six residential drug and alcohol treatment programs by 1995. One experienced public health nurse and four assistant outreach workers staff the program 15 hours each week, Monday to Friday. They provide tuberculin skin testing at some overnight shelters and at a day shelter and a clinic for the homeless. In our analysis of the program, we included all persons identified as homeless or in the residential drug/alcohol treatment programs between January 1995 and December 1998.

## Data Analysis

Persons with active TB were considered homeless if they lived in one of the shelters or residential substance-treatment centers or were reported as homeless in clinic records between 1988 and 1998. Data were available for 1995 to 1998 on whether TB diagnoses were detected by the screening program or by staff at a medical facility. We obtained the numbers of homeless persons from studies done in 1995 and 1998 by the Colorado Department of Human Services (7). These studies enumerated the persons living in shelters and residential substance treatment centers and also estimated the number of persons living in other temporary housing and on the streets. Data for homeless populations in 1996 and 1997 were not available, so we estimated this number, assuming a linear increase in the total. We analyzed TB incidence for the 11-year period, from 1988 (the year that isolates were first available for DNA fingerprinting) through 1998, compiling data for 7 years before and 4 years after the screening program was implemented for the homeless in 1995.

We analyzed identical fingerprinting patterns in homeless patients from 1988 through 1998. Initial DNA fingerprinting used the IS*6110* technique, and secondary fingerprinting used pTBN12 for isolates having five or fewer hybridizing bands (4,8). To estimate recent TB transmission in the homeless population, we calculated the proportion of cases clustered within a 2-year period of a preceding case with the identical DNA fingerprint (9). We compared the proportion of cases caused by recent transmission during the 7-year period before the program (1988–1994) with the proportion caused during the first 4 years of the program (1995–1998) to assess the effect of the screening program on TB transmission.

Analyses were done by using Epi Info, version 6.0 (Centers for Disease Control and Prevention, Atlanta, GA). The proportion clustered in the two time periods was compared by using the Fisher exact test.

## Results

### Screening Program Evaluation

The estimated number of homeless persons in Denver, Colorado, increased from 3,330 in 1995 to 5,792 in 1998. This increase was largely attributed to the combination of population growth in the metropolitan area and rising housing costs (7). The four large communal shelters could accommodate <1,176 persons and the transitional housing and treatment programs <980 persons. The number of homeless persons who had tuberculin skin tests increased from 893 in 1995 to 3,897 by 1998 (Table 1). The screening ratio (number of completed skin tests divided by the estimated population) increased from 26.7 to 67.3 per 100 persons during this period. The proportion of persons with positive tuberculin skin tests decreased during the study period (17% in 1995, 12% in 1998, p<0.01) (Table 1). Only those persons not already known to be positive were tested.

**Table 1:** Effect of screening program on tuberculin skin testing and treatment of latent tuberculosis among the homeless, Denver Health Tuberculosis Clinic, 1995–1998

Year	Tuberculin testing ratio^a^	Tuberculin skin-test positivity (%)	Completion of treatment of latent tuberculosis infection (%)
1995	893/3,350 (26.7)	150/893 (17)	7/37 (18.9)
1996	1,799/4,164 (43.2)	267/1,799 (15)	24/83 (28.9)
1997	3,438/4,978 (69.1)	397/3,438 (12)	24/93 (25.8)
1998	3,897/5,792 (67.3)	470/3,897 (12)	22/59 (37.3)

^a^Completed tuberculin tests divided by estimated homeless population (tests per 100 persons).

Active TB was diagnosed in 94 homeless persons during the 11-year period from 1988 to 1998. Of those, 87 (93%) had positive cultures. The number of confirmed cases increased to a peak of 17 cases in 1995 and then decreased to 7 during each of the next 3 years (Table 2). When the increase in the number of homeless persons during this period is considered, the estimated incidence of active TB decreased from 510 to 121 per 100,000 persons from 1995 to 1998.

**Table 2:** DNA fingerprinting results for culture-confirmed tuberculosis cases among homeless persons, Denver Health Tuberculosis Clinic, 1988–1998^a^

	Years											
RFLP clusters	1988	1989	1990	1991	1992	1993	1994	1995	1996	1997	1998	Total
A			1					1				2
B		1		[1]^b^	[2]	1						2 [3]
C	1						1				1	3
D				1		1						2
E				1	1							2
F								2				2
G				1		1				[2]		2 [2]
H			1	3 [1]	2		1			[1]		7 [2]
I			2	3	1 [2]	3 [1]	1	2 [2]	[2]			12 [7]
Total clusters	1	1	4	9 [2]	4 [4]	6 [1]	3	5 [2]	0 [2]	0 [3]	1	34 [14]
Unique RFLP	1	1	3	2	3	6	3	7	5	6	5	42
Test ratio^c^	2/4 (50)	2/2 (100)	7/7 (100)	11/11 (100)	7/7 (100)	12/12 (100)	6/6 (100)	12/17 (71)	5/7 (71)	6/7 (86)	6/7 (86)	76/87 (87)

^a^RFLP, restriction fragment length polymorphism. ^b^Numbers in brackets refer to the 14 case isolates from nonhomeless patients that share DNA fingerprinting patterns with the homeless patients. ^c^Number of case isolates with DNA fingerprinting results divided by total culture-positive cases (% tested).

The screening program was more successful in early identification of TB cases than in treatment of latent TB infection, which had low acceptance and completion rates (Table 1) that did not change substantially from 1995 to 1998. Over the 4-year period, 1,284 positive tuberculin skin tests were recorded, but only 272 homeless persons initiated isoniazid treatment; of those, 77 (28%) completed therapy. Five (29%) of the 17 confirmed cases of TB diagnosed in 1995 were detected through the screening program. Of seven TB cases diagnosed during each of the 3 subsequent years (1996–1998), the screening detected three, five and four cases, respectively, for a mean of 57%.

### DNA Fingerprinting Analysis

DNA fingerprinting results were available for 76 (87%) of the 87 culture-positive cases (Table 2). Isolates from the 76 patients demonstrated 51 different DNA fingerprinting patterns. Nine clusters, in which identical patterns occurred at any time during the study period, consisting of 2–12 patients included 34 (45%) of the 76 patients. Clusters in which cases occurred within 2 years of each other were present in six DNA fingerprinting patterns and accounted for 27 (36%). In three other clusters, occurrences of TB in patients were separated by >2 years. DNA fingerprinting patterns unique in the homeless population were found in the remaining 42 patients’ isolates, 9 of which had patterns also found in nonhomeless TB patients in the community. Of these, we detected one matching set of isolates for each of eight patterns in nonhomeless patients; one case in a homeless patient was followed 3 years later with three cases in nonhomeless persons who had isolates of the identical DNA fingerprint type (data not shown). Among the homeless patients, persons born outside the United States constituted 7 (17%) of the 42 cases with unique isolates and 3 (9%) of the 34 cases with clustered isolates.

The estimated proportion of TB cases resulting from recent transmission within the homeless population, defined as cases that were clustered within 2 years, decreased from 49% (23/47) in the 7-year period (1988–1994) before the screening program was implemented to 14% (4/29) in the 4-year period (1995–1998) after the program was implemented (p=0.03).

DNA fingerprinting results were available for 272 (55%) of 491 of culture-positive cases among persons who were not homeless. The proportion of nonhomeless case isolates with DNA fingerprinting results increased from 47% (167/353) to 53% (112/21) for the years 1988–1994 and 1995–1998, respectively. Case isolates in the nonhomeless population showed 233 DNA fingerprinting patterns with 24 clusters of two to seven patients. In four of the clusters among homeless persons, cases in homeless persons were followed by two to seven cases with the same DNA fingerprinting pattern in nonhomeless persons (Table 2). The largest cluster (I) originated with a case diagnosed in a homeless person in Denver who had stopped treatment in another state in 1990; the same strain was detected in 11 additional cases in homeless persons over a 6-year period and was also detected in 7 cases in nonhomeless persons, the first 2 of which were nosocomially acquired in 1992.

## Discussion

The institution of a TB-screening program for the homeless population in congregate settings in Denver in 1995 was associated with a substantial decrease in the incidence of active TB in this high-risk population. The mechanisms by which the program could have caused this reduction in TB incidence include treating active cases earlier and treating persons with latent TB infection, thus preventing future active TB cases. Treating persons with latent TB infection was unlikely to have played a major role, however, since we found low rates of starting and completing treatment for this type of infection, a finding noted in previous reports (1,10).

The screening program more likely reduced TB transmission by earlier diagnosis and treatment of active TB cases (the top priority activity in TB control). The program was effective in finding TB patients, detecting over half the active TB cases reported in homeless persons from 1996 to 1998. In contrast to the low acceptance and completion of latent TB infection treatment, high rates of treatment completion for active TB cases can be achieved with directly observed therapy supported by public health regulations (11,12). The results of the DNA fingerprint analysis are consistent with the conclusion that early detection of TB through the screening program leads to a reduction in transmission. New, unique strains of *M. tuberculosis* have continued to appear among the Denver homeless population, but cases from recent transmission (clustered in the homeless population within 2 years) decreased. The estimated number of active TB cases decreased despite an increasing population of homeless persons, leading to a drop in the annual incidence rate from 510 to 121 per 100,000 persons.

Other potential explanations for the reduction in TB deaths in the homeless population include failure to detect subsequent cases and errors in population estimates. The mandatory TB screening program for residents in shelters and residential substance treatment programs may have led some homeless persons to live in other settings or to leave Denver; the latter reason led to the diagnosis of subsequent cases in other jurisdictions. However, the Denver Metro TB Clinic receives reports of cases diagnosed in Metropolitan Denver and conveys them to the TB Control Program at the Colorado Department of Public Health and Environment; cases were not likely missed unless diagnosed outside Colorado. Obtaining an accurate count of homeless persons is difficult so we based our denominator in this study upon estimates obtained by the Colorado Department of Housing (7). Even if the population at risk had remained the same, despite indications otherwise, the incidence rate would still have decreased from 510 to 210 (rather than 121) per 100,000 persons per year from 1995 to 1998. We detected substantial TB transmission in the homeless population for only two of nine homeless clusters, raising the possibility that the increase in cases was the result of one or two outbreaks running their natural course. Further studies with larger numbers of cases are needed to address this issue; however, our data support the concept that early case detection results in lower rates of transmission in homeless populations.

The possibility exists that clusters that occurred over several years were strains that lingered in the community but were not detected in our study; we obtained DNA fingerprints for only 55% of the cases in the community among nonhomeless persons and cannot rule out that possibility. For clustered cases occurring many years apart, we may have missed links in transmission because of patients who were not tested in the community or who moved out of the area without diagnosis. As we increase our understanding of how to interpret the results of DNA fingerprinting, we may find that clusters that are far apart represent a remote transmission that resulted in latent TB infection with later reactivation. The secondary cases would then share the same pattern as the original (source) case, but the connection between them would be more difficult to identify since the contact in these cases occurred many years earlier.

Of the 42 homeless cases with unique patterns, seven (17%) were diagnosed in foreign-born persons. Of those, one patient arrived in United States <2 years before TB diagnosis, three 2–5 years before diagnosis, two 5–10 years before diagnosis, and one for an unknown period of time. Few of the unique patterns found among homeless patients represent cases imported from foreign countries.

Various screening programs have been proposed to decrease TB transmission among homeless persons (13). Chest radiographic screening provides a rapid assessment for potentially transmissible disease, but this method would have been too expensive for us to implement. Sputum examination by smear has been used (14) but does not detect smear-negative cases and is difficult to implement for large numbers of persons. Tuberculin skin testing does not provide an immediate assessment of infectious TB and gives negative test results for up to 25% of persons with active pulmonary TB. Furthermore, skin testing requires a second visit 2–3 days later for a reading.

Despite reaching only two thirds of the homeless population in 1998, the screening program was notably successful. The true incidence of skin test positivity is likely higher than that shown in Table 1 because those already known to be positive were not tested. The symptom survey was an important component of our program. In spite of the challenges of skin testing, our screening program proved to be feasible for a large population staying at a number of shelters and residential programs. Our results suggest that congregate settings, the focus of our program, are the most important loci of TB transmission among homeless persons.

The low completion rate of treatment for latent TB infection provided further evidence that the primary effect of the program was to increase early detection of active cases of TB, not to identify and treat latent TB infection. Despite the decrease in TB incidence following the screening program, the TB rate remained high (121 per 100,000 population) compared to the overall rate for the metropolitan area (8.2 per 100,000 population). Further progress in decreasing TB among homeless persons will require improvements in the acceptance and completion of latent infection treatment (6).

## References

[1] Barnes PF, Yang Z, Preston-Martin S, Pogoda JM, Jones BE, Otaya M, Patterns of tuberculosis transmission in central Los Angeles. JAMA. 1997;278:1159–63. 10.1001/jama.278.14.1159 9326475

[2] Nolan CM, Elarth AM, Barr H, Saeed AM, Risser DR. An outbreak of tuberculosis in a shelter for homeless men: a description of its evolution and control. Am Rev Respir Dis. 1991;143:257–61. 1990937 10.1164/ajrccm/143.2.257

[3] Moss AR, Hahn JA, Tulsky JP, Daley CL, Small PM, Hopewell PC. Tuberculosis in the homeless: a prospective study. Am J Respir Crit Care Med. 2000;162:460–4. 10934071 10.1164/ajrccm.162.2.9910055

[4] Burman WJ, Reves RR, Hawkes AP, Reitmeijer CA, Yang Z, El-Hajj H, DNA fingerprinting with two probes decreases clustering of *Mycobacterium tuberculosis.* Am J Respir Crit Care Med. 1997;155:1140–6. 9117000 10.1164/ajrccm.155.3.9117000

[5] Rendleman NJ. Mandated tuberculosis screening in a community of homeless people. Am J Prev Med. 1999;17:108–13. 10.1016/S0749-3797(99)00052-5 10490052

[6] American Thoracic Society and Centers for Disease Control and Prevention. Targeted testing and treatment of latent tuberculosis infection. Am J Respir Crit Care Med. 2000;161:S221–47. 10764341 10.1164/ajrccm.161.supplement_3.ats600

[7] D'Alanno TA. Homelessness in the Denver Metropolitan Area: a base line point in time study, June 15, 1998. The Colorado Department of Human Services. Available from: URL: http://www.cdhs.state.co.us/shhp/

[8] van Embden JD, Cave MD, Crawford JT, Dale JW, Eisenach KD, Gicquel B, Strain identification of *Mycobacterium tuberculosis* by DNA fingerprinting: recommendations for a standardized methodology. J Clin Microbiol. 1993;31:406–9. 8381814 10.1128/jcm.31.2.406-409.1993 PMC262774

[9] Jasmer RM, Hahn JA, Small PM, Daley CL, Behr MA, Moss AR, A molecular epidemiologic analysis of tuberculosis trends in San Francisco, 1991-1997. Ann Intern Med. 1999;130:971–8. 10383367 10.7326/0003-4819-130-12-199906150-00004

[10] Tulsky JP, Pilote L, Hahn JA, Zolopa AJ, Burke M, Chesney M, Adherence to isoniazid prophylaxis in the homeless: a randomized controlled trial. Arch Intern Med. 2000;160:697–702. 10.1001/archinte.160.5.697 10724056

[11] Burman WJ, Cohn DL, Rietmeijer CA, Judson FN, Sbarbaro JA, Reves RR. Noncompliance with directly-observed therapy for tuberculosis: epidemiology and effect on the outcome of treatment. Chest. 1997;111:1168–73. 10.1378/chest.111.5.1168 9149565

[12] Burman WJ, Cohn DL, Rietmeijer CA, Judson FN, Sbarbaro JA, Reves RR. Short-term incarceration for the management of noncompliance with tuberculosis treatment. Chest. 1997;112:57–62. 10.1378/chest.112.1.57 9228358

[13] Centers for Disease Control and Prevention. Prevention and control of tuberculosis among homeless persons. Recommendations of the Advisory Council for the Elimination of Tuberculosis. MMWR Recomm Rep. 1992;41(RR-5):13–23. 1314323

[14] Kimerling ME, Shakes CF, Carlisle R, Lok KH, Benjamin WH, Dunlap NE. Spot sputum screening: evaluation of an intervention in two homeless shelters. Int J Tuberc Lung Dis. 1999;3:613–9. 10423224

